# An overview of the host spectrum and distribution of *Calodium hepaticum* (syn. *Capillaria hepatica*): part 1—Muroidea

**DOI:** 10.1007/s00436-013-3691-x

**Published:** 2013-11-19

**Authors:** Hans-Peter Fuehrer

**Affiliations:** Institute of Parasitology, Department of Pathobiology, University of Veterinary Medicine Vienna, Veterinärplatz 1, 1210 Vienna, Austria

## Abstract

*Calodium hepaticum* (syn. *Capillaria hepatica*) is a worldwide-distributed species of zoonotic nematodes with a high affinity to the liver. Several rodent species of the superfamily Muroidea serve as main hosts for this pathogen. *C. hepaticum* has been found in Muroidean hosts in more than 60 countries in Europe; North, Central, and South America; Asia; Africa; and Oceania. *C. hepaticum* was documented in more than 90 Muroidean rodent species (Murinae, Deomyinae, Arvicolinae, Neotominae, Cricetinae, Sigmodontinae, Gerbillinae, and Cricetomyinae). Globally, the Norway rat (*Rattus norvegicus*) seems to be the main host species for this nematode. However, locally high prevalences (above 50 %) have also been observed in several other synanthropic (commensal and non-commensal) Muroidea species (e.g., *Rattus tanezumi*, *Ondatra zibethicus*, *Apodemus sylvaticus*). This review gives an overview of the distribution and host spectrum of *C. hepaticum* in Muroidea host species.

## Introduction

*Calodium hepaticum* (syn. *Capillaria hepatica*) is a zoonotic nematode parasite distributed worldwide. Adults of this nematode parasitize the liver of mammals and lay their eggs into the liver parenchyma causing hepatic capillariasis. The eggs are only released into the environment with the death of the host. The main hosts of this parasite are rodents of the superfamily Muroidea (Schmidt 2001). Furthermore, this parasite has been documented in numerous other mammalian species including more than 70 human cases (reviewed in Fuehrer et al. 2011; Fuehrer 2013). Hepatic capillariasis is diagnosed through necroscopy or biopsy only, because with hepatic infections eggs are not shed into the environment with the feces.

This review focuses on the Muroidea host spectrum and its geographic distribution in those hosts only. Information about the pathogenesis, ecology, and host spectrum in humans and other mammalians is given elsewhere (e.g., Fuehrer et al. 2011; Fuehrer 2013; Schmidt 2001).

For data evaluation, the systematic search was based on electronic databases (Scopus, PubMed, Google Scholar) and previous summaries (e.g., Schmidt 2001). The search terms *Capillaria hepatica*, *Calodium hepaticum*, *Hepaticola hepatica*, *Trichocephalus hepaticus*, and hepatic capillariasis were used. An attempt was made to include only those studies where the scientific names of the host and parasite were given clearly. Furthermore, spurious infections (= pseudoparasitism) were differentiated as far as possible from hepatic capillariasis. A short overview of spurious *C. hepaticum* infections in animals is given in Fuehrer (2013).

## Taxonomy

*C. hepaticum* is a nematode out of the family Capillaridae (order Trichocephalida). Moravec (1982) categorized *C. hepaticum* in the genus *Calodium*. However, the name *C. hepaticum* is rarely used, and most researchers use the term *Capillaria hepatica*. Further synonyms are *Trichocephalus hepaticus* (Bancroft, 1893) and *Hepaticola hepatica* (Hall 1916) (Fuehrer et al. 2011).

The taxonomy of the family Capillaridae is disputed and pending. In the past, most species were included in the genus *Capillaria*. Recently, a molecular phylogenetic study revealed that Capillaridae can be clearly separated from Trichuridae (Guardone et al. 2013). However, the former genus *Capillaria* consists of a complex group of parasites including several parasites of carnivores and rodents of the genera *Calodium*, *Eucoleus*, *Capillaria*, *Paracapillaria*, *Pearsonema*, and *Aonchotheca* (Guardone et al. 2013). Three species are of zoonotic importance, namely *Paracapillaria philippinensis* (syn. *Capillaria philippinensis*), *Eucoleus aerophila* (syn. *Capillaria aerophila*), and *C. hepaticum* (syn. *C. hepatica*).

## Life cycle

The life cycle of *C. hepaticum* is a direct one with a high affinity to the liver. After the ingestion of embryonated eggs, larvae hatch in the area of the caecum and invade the liver via the portal vein system. Adult worms parasitize in the liver of its mammalian hosts where the females lay eggs into the liver parenchyma after mating. The life span of adult worms is short (18–60 days post infection in mice) (Juncker-Voss et al. 2000; Schmidt 2001). The eggs develop in the host's liver to the eight-cell stage only. Unembryonated eggs are only released into the environment with the death of the host only (decay of host; excretion in feces of carnivores and omnivores or after cannibalism). Depending on the environmental conditions (e.g., humidity, temperature), eggs embryonate within 5–8 weeks. Laboratory studies revealed that embryonated eggs are viable for 25 months (reviewed in Juncker-Voss et al. 2000). The life cycle is closed when embryonated eggs are ingested from a mammalian host. The ingestion of non-embryonated eggs leads to pseudoparasitosis (= spurious infections) where the non-embryonated eggs are re-released with the feces and lead to mild symptoms only (reviewed in Fuehrer et al. 2011).

## Muroidea host spectrum

The mammalian superfamily Muroidea consists of rodents with a worldwide distribution (with the exception of Antarctica) including animals like rats, true mice, gerbils, and hamsters. Recent molecular phylogenetic studies classified the superfamily into 6 families, 19 subfamilies, around 280 genera, and over 1,300 species (e.g., Steppan et al. 2004).

The host spectrum of *C. hepaticum* in Muroidea hosts (and in other mammals) indicates very low host specificity. More than 90 species of at least 44 genera of the superfamily Muroidea (Murinae, Arvicolinae, Neotominae, Cricetinae, Sigmodontinae, Gerbilinae, and Cricetomyinae) are known as hosts of this parasite (Table 1). Of these, more than 55 species are rodents of the subfamily Murinae including the Norway rat (*Rattus norvegicus*), Black rat (*Rattus rattus*), and house mouse (*Mus musculus*). Prevalences above 50 % are regularly documented in Norway rats (*R. norvegicus*) and Tanezumi rats (*R. tanezumi*), and rarely in house mice (*M. musculus*), long-tailed field mice (*Apodemus sylvaticus*), muskrats (*Ondatra zibethicus*), and bank voles (*Myodes glareolus*). All of these species are known as (commensal or non-commensal) synanthropic species. Human hepatic capillariosis cases are associated with poor hygienic conditions and the presence of rodents (e.g., rats) (Fuehrer et al. 2011). Davis (1951) reported that *C. hepaticum* is significantly less prevalent in decreasing rat populations than in stationary or increasing populations. A study conducted in Michigan (USA) with deer mice revealed that parasite prevalences are correlated negatively with heterozygosity when the effects of population density were held constant (Meagher 1998). Meagher further hypothesizes that inbred populations are more susceptible to parasite infestations. Differences in the prevalences of *C. hepaticum* in different rodent host species are thought to be associated with different living and nutritional habits (Schmidt et al. 1998). Several authors report that *C. hepaticum* occurs in localized foci of the examined study areas (e.g., Reperant and Deplazes 2005; Stojčević et al. 2002). Furthermore, cannibalism may be an important egg-releasing mechanism and is an important source of infection in burrows. On the other hand, predation seems to be responsible for scattered foci of infection (Farhang-Azad 1977a, b; Stojčević et al. 2002). Decomposition is thought to be a less important egg-releasing mechanism. Environmental conditions (humidity and temperature) are also associated with the distribution of these pathogens (e.g., Resendes et al. 2009). The pathogenicity of *C. hepaticum* in Muroidea hosts is considered low, although experimental infections of rats and mice have been demonstrated to lead to hepatic failure and the death of the host (the host survival rate is reduced by 5–10 %) (Singleton and Chambers 1996). However, individual variations of the host's inflammatory reaction to the parasite have been reported. Furthermore, hypersensitivity is associated with repeated infections (Borucinska and Nielsen 1993).

**Table 1:** *Calodium hepaticum* in Muroidea

Classification	Species	Prevalence (%)	Country/region	References
Muridae				
Murinae	Norway rat (*Rattus norvegicus*)		USA	Childs et al. (1988); Shorb (1931); Wantland et al. (1956)
		82 % (of 86)	USA (Connecticut)	Conlogue et al. (1979)
		75 % (of 845)	USA (Maryland—Baltimore area and zoo)	Farhang-Azad (1977a)
		75 % (of 845)	USA (Maryland—Baltimore Zoo)	Farhang-Azad (1977b)
		87.9 % (176/201)	USA (Maryland, Baltimore)	Easterbrook et al. (2007)
			USA (New York)	Herman (1939)
		85.6 %	USA (Maryland)	Luttermoser (1936)
		94.1 % (of 1,460)	USA (Maryland)	Davis (1951)
			USA (North Carolina)	Harkema (1936)
			USA (District of Columbia)	Price and Chitwood (1931); Cram (1928)
			USA (Pennsylvania and Rhode Island)	Winfield (1933)
			USA (California)	Hall (1916)
		Spurious infection 6 % (of 150)	Canada (Quebec)	Firlotte (1948)
			Puerto Rico	Leon de (1964)
			Venezuela	Vogelsang and Espin (1949)
		20.1 % (51/254)	Colombia	Duque et al. (2012)
			Brazil	Araújo (1967); Galvão (1981); Chieffi et al. (1981); Ferreira and Andrade (1993)
			Brazil (Bahia)	Ferreira and Andrade (1993)
		54.1 % (13/24)	Brazil (Belém)	Moreira et al. (2013)
		30 %	Argentina (Buenos Aires)	Hancke (2011)
		33.3 % (5/15)	Chile	Torres and Gonzáles (1972); Rojas et al. (1971)
		1 case	England	Simmons and Walkey (1971)
		1 case	England (zoo)	Redrobe and Patterson-Kane (2005)
		A: 90.4 % (38/42) B + C: none of 38	England	Owen (1976)
		23 % (*n* = 44)	England	Webster and MacDonald (1995)
		60 % (of 29)	Portugal (Azores)	Roque (1989)
		20 % (of 20)	Portugal (Azores)	Cruz (2006)
		62.5 % (of 73)	Portugal	Roque et al. (1984)
		42 % (21/50)	Portugal Lisbon Zoo	Crespo (2012)
		20 %	Spain	Mascato et al. (1993); Feliu et al. (1985); Castro (1944); Gallego Berenguer (1959)
			France	Davoust et al. (1997)
			Italy	Perugia (1893)
		80 % (of 28)	Italy	Vanni (1938); Vanni (1947)
		30 % (of 100)	Italy (Pisa)	Ghelardoni (1966)
		30 % (of 50)	Italy	Casarosa and Ghelardoni (1965)
		36 % (17/49)	Italy (Milano)	Ceruti et al. (2001)
		54.55 % (of 143)	Italy (Sicily)	Milazzo et al. (2010b)
		74.6 %	Austria	Rydlo (1966)
		1 case	Austria	Frank (1977)
			Switzerland	Hörning (1966)
		16.4 % (of 864)	Belgium	Cotteleer et al. (1982)
			Former CSSR	Mituch (1960)
		100 % (26/26)	Hungary (zoo)	Mészáros and Kemenes (1973)
		1.95 % (6/307)	Croatia	Stojčević et al. (2002)
		10.9 % (of 147)	Serbia (Belgrad)	Kataranovski et al. (2010)
			Turkey	Merdivenci (1970)
			Kazakhstan	Pleščëv and Kozlov (1978)
			Japan	Shimatani (1961); Sato and Shimatani (1960); Iwaki et al. (1993); Ito et al. (1996); Yagisawa (1978)
		52.7 % (1,272/2,222)	Japan (Osaka)	Momma (1930)
		90 %	Philippines	Tubangui (1931)
		60/138 (42 %)	Thailand	Chaiyabutr (1979)
		12.5 % (of 16)	Thailand	Namue and Wongsawad (1997)
			Malaysia	Liat et al. (1977); Sinniah et al. (1979)
			China	Lagrange (1924)
		30.4 %	China (Soochow)	Wu (1930)
		7.1 %	China (Canton)	Chen (1933)
		61.9 %	China (Hubei Province)	Zhou et al. (1991)
		66.7 %	China (Yunnan Province)	Zhou et al. (1998)
		1 case	China (Yunnan Province)	Xiong et al. (1999)
		77 %	China (Yunnan Province)	Shen et al. (2003)
		66.7 %	China (Fujian Province)	Yuan et al. (2000)
		12.3 %	China (Fujian Province)	Xue et al. (1998)
		46.2 %	China (Fujian Province)	Zhang et al. (2003)
		25.8 %	China (Henan Province)	Lin et al. (2007)
		25.83 % (109/422)	China (Henan)	Wang et al. (2013)
		36.7 %	Taiwan	Yang and Lu (2000)
		54.9 %	Taiwan	Tung et al. (2009)
		62.5 % (20/32)	Taiwan	Tung et al. (2013)
		36 %	South Korea (Seoul)	Nakamura and Kobashi (1935)
		88 % (286/235)	South Korea (Seoul)	Seo et al. (1964)
		38.1 % (of 1,000)	South Korea (Seoul)	Min (1979)
		12.1 % (of 33)	South Korea (Pochun and Chungpyong)	Seo et al. (1968)
		23.6 % (21/89)	South Korea (Gangwon Province)	Yi et al. (2010)
		25.9 % (11/43)	South Korea (Chunchon)	Seong et al. (1995)
		13.04 % (of 23)	Iran	Pakdel et al. (2013)
		28 %	Australia (Queensland)	Singleton et al. (1991)
			Egypt	El-Nassery et al. (1991)
			Tunisia	Mishra and Gonzalez (1975)
	Black rat (*Rattus rattus*)		New Zealand	Roberts (1990)
		5 %	Australia (Queensland)	Singleton et al. (1991)
		25–30 %	Federated States of Micronesia (Pohnpei)	Storer (1962)
			Bangladesh	Bhuiyan et al. (1995)
			India	Chahota et al. (1997); Kumar et al. (1985); Somvanshi et al. (1995); Chahota et al. (1997); Bhattacharya et al. (1998)
		29.54 % (of 88)	India	Malsawmtluangi and Tandon (2009)
		2.32 % (1/43) Spurious infection	India	Sharma et al. (2012)
		88.3 %	India	Patel et al. (2004)
		7.3 % (of 3,190)	Pakistan	Ahmad et al. (2011)
		20 % (1/5)	Iran	Pakdel et al. (2013)
		7.4 % (2/27)	Thailand	Chaiyabutr (1979)
		4.54 % (of 22)	Thailand	Namue and Wongsawad (1997)
		28.6 %	Taiwan	Tung et al. (2009)
		18.2 % (2/11)	Taiwan	Tung et al. (2013)
			Japan	Sato and Shimatani (1960); Shimatani (1961)
			Turkey	Merdivenci (1970)
		3.1 % (2/65)	Israel	Wilamowski et al. (2002)
			Spain	Feliu et al. (1985); Castro (1944); Gallego Berenguer (1959)
			Portugal (Azores)	Casanova et al. (1996); Roque (1989)
			France	Davoust et al. (1997)
		34.2 % (of 37)	Italy (Sicily)	Milazzo et al. (2010a)
			Switzerland	Hörning (1966)
			USA	Layne (1970)
			Brazil	Chieffi et al. 1981
			Brazil (Bahia)	Ferreira and Andrade (1993)
		69.8 % (30/43)	Brazil (São Paulo)	Almeida-Silva et al. (2011)
		38.4 % (10/26)	Brazil (Belém)	Moreira et al. (2013)
			Egypt	El-Nassery et al. (1991)
		6.2 % (19/308)	Ethiopia	Farhang-Azad and Schlitter (1978)
			Democratic Republic of the Congo	Dubois (1933)
		5.8 % (6/103)	Nigeria	Onyenwe et al. (2009)
	*Rattus* spp. (*R. norvegicus* and/or *R. rattus*)	100 % (of 12)	Philippines	Claveira et al. (2005)
		34 %	Japan (Southern Anami Islands)	Kamiya et al. (1968)
		44 % (of 82)	France	Davoust et al. (1997)
	*Rattus* spp. (*Rattus rattus diardii*, *R. norvegicus*, and *R. exulans*)	21.6 %	Malaysia	Paramasvaran et al. (2009)
	*Rattus* sp.	11.9 %	France—Lyon Zoo	Apéry (2012)
		13 %	France—Vincennes Zoo	Apéry (2012)
	*Rattus rattus sladerni*	38.8 %	China (Yunnan Province)	Shen et al. (2003)
		33 % (1/3)	China (Yunnan Province)	Xiong et al. (1999)
	Polynesian rat (*Rattus exulans*)		New Zealand	Roberts (1990)
			Indonesia	Brown et al. (1975b)
			Malaysia	Liat et al. (1977); Sinniah et al. (1979)
		37.5 %	Malaysia	Syad-Arnez and Mohd Zain (2006)
	Sikkim rat (*Rattus andamanensis*)	8.3 % (1/12)	Bangladesh	Fuehrer et al. (2012)
	Rice-field rat (*Rattus argentiventer*)		Indonesia	Brown et al. (1975b)
			Malaysia	Mulkit and Cheong (1971); Liat et al. (1977); Sinniah et al. (1979)
	Lesser rice-field rat (*Rattus losea*)	5.4 %	Taiwan	Yang and Lu (2000)
		38.9 %	China (Fujian Province)	Yuan et al. (2000)
	Hoffmann's rat (*Rattus hoffmanni*)		Indonesia	Brown et al. (1975b)
	Opossum rat (*Rattus marmosurus*)		Indonesia	Brown et al. (1975b)
	Tanezumi rat (*Rattus tanezumi*)		Indonesia	Brown et al. (1975b); Wiroreno (1978)
			Malaysia	Liat et al. (1977); Sinniah et al. (1979)
	*Rattus flavipectus* (syn. for *Rattus tanezumi*)	12.9 % (20/155)	China (Henan)	Wang et al. (2013)
		12.9 %	China (Henan Province)	Lin et al. (2007)
		61.9 %	China (Hubei Province)	Zhou et al. (1991)
		65.1 %	China (Yunnan Province)	Zhou et al. (1998)
		49.4 % (of 881)	China (Yunnan Province)	Xiong et al. (1999)
		77.5 %	China (Yunnan Province)	Shen et al. (2003)
		44.3 %	China (Fujian Province)	Yuan et al. (2000)
		13.1 %	China (Fujian Province)	Xue et al. (1998)
		66.7 %	China (Fujian Province)	Zhang et al. (2003)
	Malayan field rat (*Rattus tiomanicus*)		Malaysia	Mulkit and Cheong (1971); Liat et al. (1977); Sinniah et al. (1979)
		44.4 %	Malaysia	Syad-Arnez and Mohd Zain (2006)
	Annandale's rat (*Rattus annandalei*)		Malaysia	Liat et al. (1977); Sinniah et al. (1979)
	Himalayan field rat (*Rattus nitidus*)	40.1 %	India	Malsawmtluangi and Tandon (2009)
	Bush rat (*Rattus fuscipes*)		Australia	Singleton et al. (1991); Spratt and Singleton (1986)
	Müller's giant Sunda rat (*Sundamys muelleri*)		Malaysia	Liat et al. (1977)
		33.3 %	Malaysia	Syad-Arnez and Mohd Zain (2006)
	Greater bandicoot rat (*Bandicota indica*)		Malaysia	Liat et al. (1977)
		11.5 %	Taiwan	Yang and Lu (2000)
			Sri Lanka	Dissanaike and Paramananthan (1961)
	Lesser bandicoot rat (*Bandicota bengalensis*)		Bangladesh	Bhuiyan et al. (1995)
			India	Pasricha et al. (1941)
		33.3 % (6/18)	India	Singla et al. (2013)
	Bower's white-toothed rat (*Berylmys bowersi*)		Malaysia	Liat et al. (1977)
		16.6 %	India	Malsawmtluangi and Tandon (2009)
	Kenneth's white-toothed rat (*Berylmys mackenziei*)	31.8 %	India	Malsawmtluangi and Tandon (2009)
	Gray tree rat (*Lenothrix canus*)		Malaysia	Liat et al. (1977)
	White-bellied rat (*Niviventer niviventer*)		Indonesia	Brown et al. (1975b)
	Chestnut white-bellied rat (*Niviventer fulvescens*)		Malaysia	Liat et al. (1977)
		40 %	India	Malsawmtluangi and Tandon (2009)
		55.6 %	China (Fujian Province)	Yuan et al. (2000)
	Dark-tailed tree rat (*Niviventer cremoriventer*)		Malaysia	Mulkit and Cheong (1971)
	Chinese white-bellied rat (*Niviventer confucianus*)	30 %	China (Fujian Province)	Yuan et al. (2000)
	*Rattus nivivente* (sug. syn. for *Niviventer* sp.)	6.12 % (3/49)	China (Henan)	Wang et al. (2013)
	Edwards's long-tailed giant rat (*Leopoldamys edwardsi*)		Indonesia	Brown et al. (1975b)
			Malaysia	Liat et al. (1977)
	Long-tailed giant rat (*Leopoldamys sabanus*)		Indonesia	Brown et al. (1975b)
			Malaysia	Mulkit and Cheong (1971); Liat et al. (1977)
	Bartels's spiny rat (*Maxomys bartelsii*)		Indonesia	Brown et al. (1975b); Wiroreno (1978)
	Hellwald's spiny rat (*Maxomys hellwaldii*)		Indonesia	Brown et al. (1975b)
	Rajah spiny rat (*Maxomys rajah*)		Malaysia	Mulkit and Cheong (1971); Liat et al. (1977)
		30.6 %	Malaysia	Syed-Arnez and Mohd Zain 2006
	Musschenbroek's spiny rat (*Maxomys musschenbroekii*)		Indonesia	Brown et al. (1975b)
	Whitehead's spiny rat (*Maxomys whiteheadi*)		Malaysia	Mulkit and Cheong (1971); Liat et al. (1977)
		25 %	Malaysia	Syed-Arnez and Mohd Zain 2006
	Red spiny rat (*Maxomys surifer*)		Malaysia	Liat et al. (1977)
		30.4 %	Malaysia	(Syed-Arnez and Mohd Zain 2006)
	Fawn-footed mosaic-tailed rat (*Melomys cervinipes*)		Australia	Singleton et al. (1991); Spratt and Singleton (1986)
	Giant white-tailed rat (*Uromys caudimaculatus*)	24 %	Australia	Singleton et al. (1991)
	Kaiser's rock rat (*Aethomys kaiseri*)		Rwanda	Fain (1955)
	Hinde's rock rat (*Aethomys hindei*)		Democratic Republic of the Congo	Fain (1953)
	Peters's striped mouse (*Hybomys univittatus*)		Democratic Republic of the Congo	Schwetz (1956)
	African grass rat (*Arvicanthis niloticus*)		Democratic Republic of the Congo	Fain (1953)
	African marsh rat (*Dasymys incomtus*)		Democratic Republic of the Congo	Fain (1953); Schwetz (1956)
	House mouse (*Mus musculus*)	6.2 %	Spain	Mascato et al. (1993); Feliu et al. (1985); Castro (1944); Gallego Berenguer (1959)
		2.6 % (1/39)	Israel	Wilamowski et al. (2002)
		9.1 % (of 22)	Russia	Romašov (1983)
			Russia	Romašov (1996)
			Kazakhstan	Pleščëv and Kozlov (1978)
			Turkey	Merdivenci (1970)
		47.4 %	Austria	Juncker et al. (1998)
		42.7 % (of 166)	Austria (Vienna—zoo)	Juncker-Voss et al. (2000)
			Switzerland	Hörning (1966)
		80 % (of 5)	Italy	Vanni (1947)
		5.5 % (of 37)	Italy (Sicily)	Milazzo et al. (2010a)
		21.2 % (of 52)	Portugal (Azores)	Casanova et al. (1996)
		19.6 % (10/51)	Portugal (Azores)	Resendes et al. (2009)
		40.2 % (of 92)	Portugal (Azores)	Pereira (2009)
		22 % (11/50)	Portugal Lisbon Zoo	Crespo (2012)
			USA	Childs et al. (1988)
			USA (Maryland)	Luttermoser (1938)
			USA (Pennsylvania)	Doran (1955)
		0.9 % (of 110)	Iran	Pakdel et al. (2013)
		4.6 % (of 410)	Pakistan	Ahmad et al. (2011)
		2.1 % (1/47)	Bangladesh	Fuehrer et al. (2012)
			Bangladesh	Bhuiyan et al. (1995)
		19.1 %	China (Hubei Province)	Zhou et al. (1991)
		21.1 %	China (Yunnan Province)	Zhou et al. (1998)
		4.6 %	China (Fujian Province)	Xue et al. (1998)
		10 %	China (Henan Province)	Lin et al. (2007)
		10 % (13/130)	China (Henan)	Wang et al. (2013)
			Australia (Queensland)	Singleton et al. (1991)
			Australia release study	Singleton and Chambers (1996)
	Long-tailed field mouse (*Apodemus sylvaticus*)	2/17	Austria	Frank (1977)
			Switzerland	Hörning (1966)
		7 % (of 99)	Switzerland (Geneva Canton)	Reperant and Deplazes (2005)
			Belgium	Bernard (1961)
			Former UDSSR	Pavlov (1955)
			Spain	Feliu et al. (1984, 1985, 1987); Mas-Coma and Feliu (1977); Prokopič and Tenora (1975)
			England	Baylis (1931)
		75 % (of 58)	England	Canning et al. (1973)
		100 %	St. Kilda, UK	Berry and Tricker (1969)
		18 % (2/11)	UK Shetland Islands	Wilson et al. (1998)
			Wales	Lewis (1968)
			Slovakia	Mituch (1966/1970)
			Bulgaria	Genov (1984); Prokopič and Genov (1974)
			Russia	Romašov (1996)
			Georgia	Kirschenblat (1948)
			Armenia	Kirakosjan et al. (1963)
			Middle Asia	Tokobaev (1976)
	Yellow-necked mouse (*Apodemus flavicollis*)		Russia	Romašov (1978, 1996)
		5.93 % (of 135)	Russia	Romašov (1983)
			Bulgaria	Genov (1984); Prokopič and Genov (1974)
		2 cases	Serbia	Ĉabrilo et al. (2013)
			Slovakia	Mituch (1960); Mituch (1966/1970)
			Former CSSR	Erhardová (1956); Erhardová and Ryšavy (1955); Prokopič and Genov (1974); Tenora (1963)
		8.5 % (24/284)	Germany (Saxony-Anhalt)	Schmidt (2001)
		6 cases	Denmark	Tenora et al. (1991)
	*Apodemus* spp.	1.5 % (of 96)	France (forested area near Dijon)	Scandola et al. (2013)
			Iran	Mobedi and Arfaa (1971)
	Broad-toothed field mouse (*Apodemus mystacinus*)		Georgia	Kirschenblat (1948)
	Striped field mouse (*Apodemus agrarius*)		Russia	Romašov (1978)
		3.37 % (of 297)	Russia	Romašov (1983)
		0.2 %	Russia (Southern West Siberia)	Chechulin et al. (2011)
			Former UDSSR	Pavlov (1955)
			Russia (Novosibirsk Region)	Koval'chuk and Bonina (1981)
		4.27 % (5/117)	China (Henan)	Wang et al. (2013)
	Small Japanese field mouse (*Apodemus argenteus*)		Japan	Chabaud et al. (1963); Ishimoto (1974); Iwaki et al. (1993)
	Korean field mouse (*Apodemus peninsulae*)		Japan	Iwaki et al. (1993)
	Large Japanese field mouse (*Apodemus speciosus*)		Japan	Iwaki et al. (1993)
	Typical striped grass mouse (*Lemniscomys striatus*)		Democratic Republic of the Congo	Fain (1953)
	Southern multimammate mouse (*Mastomys coucha*)		Democratic Republic of the Congo	Fain (1953); Schwetz (1956)
	Natal multimammate mouse (*Mastomys natalensis*)		Ghana	Paperna et al. (1970)
			South Africa	Cochrane et al. (1957)
	Jackson's soft-furred mouse (*Praomys jacksoni*)		Democratic Republic of the Congo	Fain (1953)
	Tropical Vlei rat (*Otomys tropicalis*)		Democratic Republic of the Congo	Fain (1953)
	Creek groove-toothed swamp rat (*Pelomys fallax*)		Democratic Republic of the Congo	Schwetz (1956)
	Bell groove-toothed swamp rat (*Pelomys campanae*)		Guinea	Joyeux et al. (1928)
	Target rat (*Stochomys longicaudatus*)		Democratic Republic of the Congo	Schwetz (1956)
	Ethiopian white-footed mouse (*Stenocephalemys albipes*)	0.5 % (1/212)	Ethiopia	Farhang-Azad and Schlitter (1978)
Deomyinae	Yellow-spotted brush-furred rat (*Lophuromys flavopunctatus*)		Democratic Republic of the Congo	Schwetz (1956)
	Southern African spiny mouse (*Acomys spinosissimus*)		Zimbabwe	Sandground (1933)
Cricetidae				
Arvicolinae				
	Bank vole (*Myodes glareolus*)		Russia	Romašov (1978, 1996)
		37.36 % (of 1,159)	Russia	Romašov (1983)
		1.4 %	Russia (Southern West Siberia)	Chechulin et al. (2011)
			Former UDSSR	Pavlov (1955)
		75 % (of 57)	England	Canning et al. (1973)
		27.6 % (of 29)	France (forested area near Dijon)	Scandola et al. (2013)
		15.1 % (22/146)	Germany (Saxony-Anhalt)	Schmidt et al. (1998); Schmidt (2001)
		5.2 % (of 58)	Switzerland (Geneva Canton)	Reperant and Deplazes (2005)
			Slovakia	Mituch (1960)
		5.4 % (of 115)	Czech Republic	Rupeš (1964)
	Northern red-backed vole (*Myodes rutilus*)		Former UDSSR	Pavlov (1955)
		1 %	Russia (Southern West Siberia)	Chechulin et al. (2011)
	Southern red-backed vole (*Myodes gapperi*)		USA	Fisher (1963)
		9.5 % (28/294)	USA	Solomon and Handley (1971)
		2.8 %	Canada (Alonquin Park)	Freeman and Wright (1960)
	Grey red-backed vole (*Myodes rufocanus*)		Japan	Chabaud et al. (1963); Ishimoto (1974); Iwaki et al. (1993)
	Northern mole vole (*Ellobius talpinus*)		Former UDSSR	Pavlov (1955)
	Zaisan mole vole (*Ellobius tancrei*)		???	Mentioned in Tinnin et al. (2011)
	Siberian brown lemming (*Lemmus sibiricus*)		Former UDSSR	Morozow (1956)
			USA	Rausch (1961)
	Southern bog lemming (*Synaptomys cooperi*)		Canada (Alonquin Park)	Freeman and Wright (1960)
	Muskrat (*Ondatra zibethicus*)		Canada (Alonquin Park)	Freeman and Wright (1960)
			Canada (Ontario)	Price (1931)
		Laboratory infection studies	USA	Borucinska et al. (1997)
		77 % (184/270)	USA (Pennsylvania and Connecticut)	Borucinska et al. (1993)
			USA (Louisiana)	Penn (1952)
		17 % (of 104)	USA (Maine)	Meyers and Reilly (1950)
			USA (Michigan)	Ameel (1942)
			Russia	Romašov (1995, 1996)
			Former CSSR	Tenora and Zavadil (1967)
		4.21 % (of 1,140)	Belgium	Cotteleer et al. (1982)
		1 case (of 440)	Great Britain	Warwick (1937)
	Field vole (*Microtus agrestis*)	3 cases (of 5)	Austria	Frank (1977)
		16.67 % (of 6)	Russia	Romašov (1983)
			Russia	Romašov (1978, 1996)
		4.5 %	Russia (Southern West Siberia)	Chechulin et al. (2011)
	Common vole (*Microtus arvalis*)	0.9 % (3/318)	Austria	Fuehrer et al. (2010)
		4 cases (of 4)	Austria	Frank (1977)
		20.69 % (of 29)	Russia	Romašov (1983)
			Russia	Romašov (1996)
	Rock vole (*Microtus chrotorrhinus*)		USA	Fisher (1963)
			Canada	Freeman and Wright (1960); Lubinsky et al. (1971)
	Meadow vole (*Microtus pennsylvanicus*)		Canada	Lubinsky et al. (1971)
		9.4 % (of 769)	Canada (Alonquin Park)	Freeman and Wright (1960)
	Tundra vole (*Microtus oeconomus*)		Former UDSSR	Morozow (1956)
		3.4 %	Russia (Southern West Siberia)	Chechulin et al. (2011)
			Canada	Freeman and Wright (1960)
	Narrow-headed vole (*Microtus gregalis*)		Kyrgyzstan	Tokobaev (1960)
	Günther's vole (*Microtus guentheri*)	1 case	England (zoo)	Redrobe and Patterson-Kane (2005)
	Water vole (*Arvicola terrestris*)	1.1 % (1/98)	Austria	Fuehrer et al. (2010)
			Russia	(Chechulin 1989); Romašov (1978, 1996)
		10.4 %	Russia (Southern West Siberia)	Chechulin et al. (2011)
		28.57 % (of 42)	Russia	Romašov (1983)
			Switzerland	Hörning (1966)
		0.2 % (of 466)	Switzerland (Geneva Canton)	Reperant and Deplazes (2005)
		2 cases	England (zoo)	Redrobe and Patterson-Kane (2005)
	European snow vole (*Chionomys nivalis*)		Former UDDSR	Pavlov (1955)
			Former UDDSR	Kirschenblatt (1938)
	Brandt's vole (*Lasiopodomys brandtii*)		China (Inner Mongolia)	Wan et al. (2007a)
Neotominae	Eastern wood rat (*Neotoma floridana*)	47.1 % (16/34)	USA	Solomon and Handley (1971)
	Bushy-tailed woodrat (*Neotoma cinerea*)		USA	Rausch (1961)
	Cotton mouse (*Peromyscus gossypinus*)		USA	Layne (1968, 1970); Layne and Winegarner (1971)
	White-footed mouse (*Peromyscus leucopus*)	2.9 % (7/239)	USA	Solomon and Handley (1971)
	Deer mouse (*Peromyscus maniculatus*)	10.2 % (73/713)	USA	Solomon and Handley (1971)
			USA (lab experiments)	Meagher (1998)
			Canada	Lubinsky (1957); Lubinsky et al. (1971); Freeman and Wright (1960); Freeman (1958); Wright (1961); Herman (1981)
			Canada (Alberta)	Lubinsky (1956)
	Florida mouse (*Podomys floridanus*)		USA	Rausch (1961); Layne (1968, 1970); Layne and Winegarner (1971)
		12.7 % (21/723)	USA (Florida)	Layne and Griffo Jr (1961)
	*Reithrodontomys* sp.		USA	King and Stanton (1974)
Cricetinae	Gray dwarf hamster (*Cricetulus migratorius*)		Former UDSSR	Pavlov (1955)
	European hamster (*Cricetus cricetus*)		Austria	Frank (1977)
	Greater long-tailed hamster (*Tscherskia triton*)		China (Henan)	Wang et al. (2013)
	Campbell's dwarf hamster (*Phodopus campbelli*)		China (Inner Mongolia)	Wan et al. (2007a, b)
Sigmodontinae	Northern grass mouse (*Necromys urichi*)		Venezuela	Vogelsang and Espin (1949)
	Hispid cotton rat (*Sigmodon hispidus*)		USA	Luttermoser (1937); Layne (1968, 1970)
			USA (Texas)	Read (1949)
		Freshwater marshes: 30 % (43/142); salt water marshes 12 % (4/34); upland habitats 5 % (1/22)	USA (Florida)	Kinsella (1974)
Gerbillinae	Savanna gerbil (*Gerbilliscus validus*)		Democratic Republic of the Congo	Fain (1953)
			Democratic Republic of the Congo	Schwetz (1956)
	Bushveld gerbil (*Gerbilliscus leucogaster*)		Democratic Republic of the Congo	Schwetz (1956)
	Persian jird (*Meriones persicus*)		Armenia	Kirakosjan et al. (1963)
		6.9 % (11/160)	Iran	Kia et al. (2010)
Cricetomyinae	Emin's pouched rat (*Cricetomys emini*)	17.7 %	Democratic Republic of the Congo	Malekani (1990), 1994)
			Rwanda	Fain (1955)
	Gambian pouched rat (*Cricetomys gambianus*)	30.8 %	Democratic Republic of the Congo	Malekani (1990), 1994)
			Nigeria	Chineme and Ibrahim (1984)

## Hepatic capillariasis—geographic distribution in Muroidea hosts

*C. hepaticum* has been found in Muroidean hosts in more than 60 countries in Europe; North, Central, and South America; Asia; Africa; and Oceania. *R. norvegicus* is the rodent species with the highest prevalences worldwide. In Europe, North America, South America, and Asia, several studies reported prevalences above 50 % in Norway rats (e.g., Easterbrook et al. 2007). Also other murid host species can present high prevalences in certain regions. In Asia, the nematode was found in prevalences above 50 % in the common species *R. tanezumi* and the white bellied rat (*Niviventer fuloscens*) (e.g., Yuan et al. 2000; Zhou et al. 1998). Furthermore, the muskrat (*O. zibethicus*) seems to be an important host of *C. hepaticum* in North America (Borucinska and Nielsen 1993). In the UK, high prevalences of this parasite were observed in long-tailed field mice (*A. sylvaticus*) and the bank vole (*M. glareolus*) (Canning et al. 1973).

## Conclusions

*C. hepaticum* is a worldwide-distributed parasite with rodents of the superfamily Muroidea as main hosts. *C. hepaticum* has been described in more than 90 rodent species. Murinae and Arvicolinae are the hosts with the highest prevalences of this parasite. The Norway rat seems to be the most important host species with reported prevalences above 50 % on several continents. However, a high percentage of the studies dealt with Norway rats only, and not with less common murid rodents. Especially synanthropic (commensal and non-commensal) Murinae and Arvicolinae seem to be the most affected hosts.

However, the diagnosis of this pathogen is limited to liver biopsies and necroscopy and so the true prevalence in Muroidea and other mammals remains unclear. At spurious infections, care should be taken to exclude mix-ups with other Trichuridae and Capillaridae shedding eggs of almost similar morphology (e.g., Bork-Mimm and Rinder 2011; Di Cesare et al. 2011; Stuart et al. 2013; Traversa et al. 2011). Novel (molecular) diagnostic tools for proper (molecular) species classification are of urgent need.
